# Detection and follow-up of chronic obstructive pulmonary disease (COPD) and risk factors in the Southern Cone of Latin America. the pulmonary risk in South America (PRISA) study

**DOI:** 10.1186/1471-2466-11-34

**Published:** 2011-06-01

**Authors:** Adolfo L Rubinstein, Vilma E Irazola, Lydia A Bazzano, Edgardo Sobrino, Matías Calandrelli, Fernando Lanas, Alison G Lee, Jose A Manfredi, Héctor Olivera, Jacqueline Ponzo, Pamela Seron, Jiang He

**Affiliations:** 1 Centro de Excelencia en Salud Cardiovascular para el Cono Sur, Instituto de Efectividad Clínica y Sanitaria, (Emilio Ravignani 2024), Buenos Aires, (C1414CPV), Argentina; 2 Department of Epidemiology School of Public Health and Tropical Medicine, Tulane University, (1440 Canal Street), New Orleans (70112), USA; 3 Department of Cardiology, Sanatorio San Carlos, (Mitre 124), San Carlos de Bariloche, (8400) Argentina; 4 Universidad de La Frontera, (Avenida Francisco Salazar 01145), Temuco, Chile; 5 Fogarty Fellow, Centro de Excelencia en Salud Cardiovascular para el Cono Sur, Instituto de Efectividad Clínica y Sanitaria, (Emilio Ravignani 2024), Buenos Aires, (C1414CPV), Argentina; 6 Department of Cardiology, Universidad de la República, Canelones, (90000), Uruguay; 7 Secretaría de Salud, Municipalidad de Marcos Paz, Marcos Paz, (1727), Argentina; 8 Department of Family Medicine, Universidad de la República, Montevideo, (11000), Uruguay

**Keywords:** Chronic Obstructive Pulmonary Disease, Risk Factors, South America, Cohort

## Abstract

**Background:**

The World Health Organization has estimated that by 2030, chronic obstructive pulmonary disease will be the third leading cause of death worldwide. Most knowledge of chronic obstructive pulmonary disease is based on studies performed in Europe or North America and little is known about the prevalence, patient characteristics and change in lung function over time in patients in developing countries, such as those of Latin America. This lack of knowledge is in sharp contrast to the high levels of tobacco consumption and exposure to biomass fuels exhibited in Latin America, both major risk factors for the development of chronic obstructive pulmonary disease. Studies have also demonstrated that most Latin American physicians frequently do not follow international chronic obstructive pulmonary disease diagnostic and treatment guidelines. The PRISA Study will expand the current knowledge regarding chronic obstructive pulmonary disease and risk factors in Argentina, Chile and Uruguay to inform policy makers and health professionals on the best policies and practices to address this condition.

**Methods/Design:**

PRISA is an observational, prospective cohort study with at least four years of follow-up. In the first year, PRISA has employed a randomized three-staged stratified cluster sampling strategy to identify 6,000 subjects from Marcos Paz and Bariloche, Argentina, Temuco, Chile, and Canelones, Uruguay. Information, such as comorbidities, socioeconomic status and tobacco and biomass exposure, will be collected and spirometry, anthropometric measurements, blood sampling and electrocardiogram will be performed. In year four, subjects will have repeat measurements taken.

**Discussion:**

There is no longitudinal data on chronic obstructive pulmonary disease incidence and risk factors in the southern cone of Latin America, therefore this population-based prospective cohort study will fill knowledge gaps in the prevalence and incidence of chronic obstructive pulmonary disease, patient characteristics and changes in lung function over time as well as quality of life and health care resource utilization. Information gathered during the PRISA Study will inform public health interventions and prevention practices to reduce risk of COPD in the region.

## Background

Although chronic pulmonary diseases, including chronic obstructive pulmonary disease (COPD), are believed to be one of the leading causes of morbidity and mortality in the Southern cone, few studies have described the prevelance and patient characteristics in this region and no study has described COPD trends over time. The World Health Organization (WHO) has predicted a rise in the mortality from COPD, from the fourth leading cause of death in 2004 to the third in 2030, largely attributable to rising tobacco consumption and biomass fuel exposure, both major risk factors for COPD and overwhelmongly prevalent in developing countries[1-3].

Variable definitions, under-recognition and under-diagnosis of COPD and its exacerbations have made it difficult to quantify prevalence, morbidity and mortality based on available data, particularly in low and middle-income countries[4,5]. An accurate estimate of the worldwide burden of COPD remains unknown[6,7]. The Latin American Project for the Investigation of Obstructive Lung Disease (PLATINO) examined the prevalence of COPD by pre- and post-bronchodilator spirometry among persons over age 40 in five major Latin American cities and found that the overall prevalence (95% CI) of COPD varied from 7.8% (5.9-9.7) in Mexico City to 19.7% (17.2-22.1) in Montevideo and was notably higher in older subjects and males. The reasons for the range of prevalence are unclear[8]. Outside of this study, the reliability of COPD prevalence estimates is questionable. For example, there are no population-based studies of COPD prevalence in Argentina, but estimated prevalences range from 5 to 8%[9]. Interestingly, these numbers are lower than the prevalences demonstrated by the PLATINO study, and Argentine adults have among the highest mean tobacco consumption in the region (58.1 pack-years)[5].

Moreover, studies have shown rates of tobacco consumption amongst younger generations in Latin America to be shocking. For example, in Argentina, Chile and Uruguay, 25.8%, 39.2% and 22.9%, respectively, of 15-18 years olds smoke compared with WHO estimates of 13.2% of people aged 15-18 worldwide[10-12]. These numbers support the idea that Latin America is early in its COPD epidemic and the prevalence and associated costs will only continue to rise as populations age, unless appropriate preventive and treatment measures are instituted.

In 2010, the National Heart, Lung and Blood Institute (NHLBI) funded an observational prospective cohort study, led by the Instituto de Efectividad Clinica y Sanitaria (IECS) in Buenos Aires, Argentina in parnership with Tulane University School of Public Health and Tropical Medicine in New Orleans, Louisiana, U.S.A, the Universidad de la Frontera in Temuco, Chile, and the Universidad de la República, in Montevideo, Uruguay, to determine the prevalence and incidence of COPD and describe patient characteristics and change in lung function over time, as well as quality of life and health care resources utilization. Spirometry will be performed and questionnaires administered to 6,000 subjects from Marcos Paz and Bariloche, Argentina, Temuco, Chile, and Canelones, Uruguay to further knowledge of COPD in these mid-sized cities representing the Southern cone of Latin America.

## Methods/Design

The PRISA study is an observational, prospective cohort study with at least 4 years of follow-up. Subjects 45-75 years of age are being recruited using a multistage cluster sampling method. The study is composed of two phases. In the first phase, baseline data will be collected regarding exposure to risk factors and prevalence of COPD. In the second phase, annual follow-up data will be obtained to determine the rate of incident COPD, the association between exposure and development of COPD, and the rate of decline in lung function exhibited by COPD subjects identified in the first phase. Inclusion criteria were men and women aged 45-75 years of age, permanent resident at one of the study locations for at least six months each year and willingness to offer written consent. Exclusion criteria were an expressed intenton to relocate within the next four years, inability to respond to the questionnaire due to cognitive impairment or language difficulty, active tuberculosis, pregnancy, or history of detached retina and cardiac infarction or ocular, thoracic or abdominal surgery within prior six weeks (contraindications to spirometry testing). A total of 6,000 non-institutionalized, mainly urban, (1,500 participants per site), men and women will be recruited from Bariloche and Marcos Paz, Argentina, Temuco, Chile, and Canelones, Uruguay.

### Sampling Procedure

**Figure 1:**
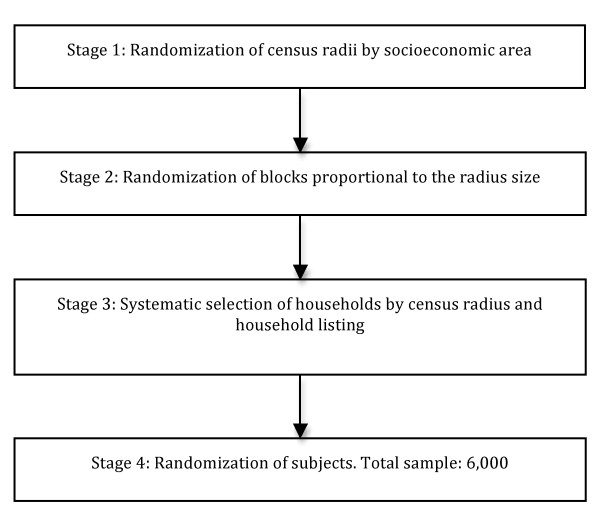
**Sampling procedure**.

A randomized, three-staged, stratified, cluster sampling strategy was usedin order to select a representative sample of the general population aged 45 to 75 years of age. The first stage selected 60 census enumeration areas using probability proportional to size, with implicit stratification by socioeconomic level of the enumeration area. The second stage selected 40 households from each enumeration area using systematic sampling. The third stage selected one household member between 45-75 years of age for the study. The final sampling was stratified by gender, with 50% men and 50% women, and age distribution based on recent population census data[13,14](Figure 1).

### Recruitment Plan

Participants identified by the sampling procedure are invited to participate through a letter from the site institution. The interviewer makes contact with the household and arranges a home visit, to collect questionnaire data, and a clinic visit. At the clinic visit, physical measurements, pre and post-bronchodilator spirometry and electrocardiogram (EKG) are performed and overnight, fasting blood samples are collected. To minimize non-participation and the potential for bias in the results, specific measures, such as travel assistance or home assessment, flexibility of appointment times, feedback of blood test and examination results and participation of local health professionals, non-governmental organizations and local authorities, have been involved to facilitate and encourage the participation of those selected for the study.

### Primary Outcome

The primary outcome for phase one of the study is the prevalence of COPD, as defined by the Global initiative for Obstructive Lung Disease (GOLD). The lower limit of normal (LLN) values for these populations will be calculated, using the American Thoracic Society (ATS) and European Respiratory Society (ERS) guidelines. The primary outcome for the four-year follow-up is the change in lung function. Secondarily, the incidence of COPD will be calculated. These criteria are listed in Table 1.

**Table 1:** Spirometric criteria for the definition of COPD

Spirometry Criteria	Definition
GOLD[1]	Post-bronchodilator forced expiratory volume in the first second (FEV1)/forced vital capacity (FVC) < 70 and FEV1 < 80% of predicted value
ATS[28,29]	Post-bronchodilator FEV1/FVC below the LLN and FEV1 < 100% of predicted value
ERS[30]	Post-bronchodilator FEV1/FVC < 88% of the predicted value for men and < 89% for women

### Secondary Measures

The following measures will also be collected: socio-demographic data including age, gender, education, race, occupation, family income; type of health coverage and degree of health service utilization; history and current status of tobacco, biomass fuel or occupational exposures; level of alcohol consumption; physical activity including type, frequency and intensity; nutritional intake; self-reported past medical history; pharmacologic and non-pharmacologic treatment received for prior medical history; self-reported family history and history of hospital admissions due to pulmonary disease during childhood. Blood pressure and anthropometrics, including height, weight, abdominal and hip circumference, will also be measured. Biochemical measurements such as lipid profile, serum creatinine and glucose will be conducted and EKG will be performed.

### Instruments

Information about current and former cigarette smoking, including age at which smoking was initiated, years of smoking, amount of cigarettes smoked per day, cessation attempts and treatments will be assessed by the Global Adult Tobacco Survey (GATS)[15]. Use of other forms of tobacco, exposure to passive cigarette smoking and indoor pollution will also be assessed using other questionnaires to assess indoor heating and cooking using biomass fuels. Nutritional information will be collected using a semi-quantitative food frequency questionnaire (FFQ) adapted from the National Cancer Institute validated by our research team to be used in Argentina, Chile and Uruguay[16]. Depression and anxiety will be assessed by the nine item Patient Health Questionnaire (PHQ-9)[17], which has been validated in Argentina[18]. Traumatic, stressful events and spirituality will be assessed through the Hispanic Community Health Study/Study of Latinos (HCHS/SOL) study questionnaire which has been cross-culturally adapted[19]. The International Physical Activity Questionnaire (IPAQ)[20] will be used to assess physical activity. SF-12[21] and European Quality of Life 5-Dimensions (EQ5D)[22] questionnaires will be used to evaluate health-related quality of life and social preferences, respectively through locally validated versions of these tools[23,24]. The remainder of the questionnaires used to gather participant information have been cross-culturally adapted from the HCHS/SOL [19].

Spirometric measurements will be performed with identical, portable, battery operated, ultrasound transit-time based EasyOne^™ ^spirometers (Medical Technologies, Chelmsford, Massachusetts and Zürich, Switzerland). Calibration will be checked daily with a three-liter syringe. Test results will be stored in the spirometer memory and downloaded weekly to a central computer. Trained professionals will first administer a questionnaire to determine patient eligibility, as described above. Trained personnel will then perform spirometry following ATS guidelines. Spirometry will be conducted with subjects in the seated position, wearing a nose clip and using a disposable mouthpiece. Subjects will perform up to eight forced expiratory maneuvers with forced vital capacity (FVC) and forced expiratory volume in the first second (FEV1) reproducible within 150 ml. A beta-agonist bronchodilator, Albuterol 200 ug, will be administered and repeat spirometry will be performed 15 minutes later, using the same criteria. All field methods have been tested in pilot studies at each site.

All health care professionals involved in the study have participated in a two-day spirometry and questionnaire training led by the main study coordinator, a pulmonologist from the coordinating center (E.S.). The spirometry course focused on the acceptability and reproducibility of a pulmonary function test and the factors associated with an unacceptable spirometry test. The technicians were certified if they were able to perform 10 pulmonary function tests that met the 2005 ATS/ERS criteria.

## Statistical Analysis

### Sample size

The calculated sample size is 6,000 participants (1,500 per site). This sample will be sufficient to provide precise estimates of the prevalence of COPD by gender and site and associated risk factors, in three age-defined categories: 45 to 54, 55 to 64 and 65 to 75 years old. The proposed sample size is sufficient to comply with the precision requirements of a complex sample that assumes a design effect of 1.5 and the prevalence of risk factors of interest of 5% or greater (Table 2). All power calculations used a statistically significant alpha level of 0.05 and a statistical power of 85%, which will permit detection of moderate and large relative risks.

**Table 2:** Sample size for a highly complex study design for effect and design of specific proportion

Proportion	Design Effect				
	1.0	1.5	2.0	2.5	3.0

0.26-0.50	30	45	60	75	90

0.25	32	48	64	80	96

0.20	40	60	80	100	120

0.15	53	80	107	133	160

0.10	80	120	160	200	240

0.05	160	240	320	400	480

### Statistical Analysis

General characteristics of the population will be described. For continuous variables, mean and median, range, standard deviation, and/or quartile range will be calculated according to the distribution of each variable. In the case of categorical variables, absolute and relative frequencies will be calculated.

In order to determine the prevalence and incidence of COPD and patient characteristics and risk factors, the design effect of the first stage unit of sampling will be considered. Weighting will be based on the relation between the number of individuals included in the study and the population size and composition of each site according to the most recent census data. Likewise, the analysis will be carried out by socioeconomic strata, gender and age categories.

To assess the association between risk factors and COPD, linear regression and simple and multiple logistical regressions will be used according to the nature of the response variables. Continuous variables that are not normally distributed will be evaluated by the application of transformations and categorizations wherever applicable[25]. The secular trends in risk factors over time will be evaluated with methods of statistical analysis that take into account the correlation between repeated measures. To evaluate the changes in risk factors over time by sub-groups of interest, generalized estimation equations will be used. To estimate the rate of accumulated COPD incidents, the Kaplan-Meir method will be used. The log rank test will be used to compare the differences between the curves of accumulated incidence events. The log rank test of trend will be applied to analyze the relationship between the accumulated risk by quartile group or by created groups of interest. In order to quantify the relationship between risk factors and the incidence of COPD, the Cox Proportional Hazards method will be used. Potential confounders and interactions will be explored.

Appropriate diagnostics will be carried out to test goodness of fit, co-linearity, and atypical observations in each model. In all cases, fulfillment of assumptions in the model by means of exploration of residual behavior will be verified. Statistical analysis software STATA 10.0 and SAS 9.0 will be used[26,27].

## Ethical Considerations

The protocol has obtained formal ethical approval from the respective Institutional Review Boards in Argentina, Chile, Uruguay, and USA. The PRISA study will strictly follow guidelines for the protection of the rights of human volunteers. All investigators and personnel in the study have attended a training session regarding this theme, certified by the NIH. All participants will sign the informed consent during the initial visit. To protect participant confidentiality, the information included in database will not contain personal information. Each participant in the study will be assigned a unique identification code (ID), which will not be a mathematical derivation of the medical registration of the patient or any other personal identifier.

All transmission of data obtained in the field, along with the central lab results and the coordination of the data will take place via a secure website requiring a password for access. Blood samples will be stickered with the ID only, to be transmitted and processed in the central lab. No personal information of any type will be in any presentation or publication. In addition, all study personnel will sign a confidentiality agreement to not divulge information or data related to the study.

## Discussion

Despite the burden of COPD in Latin America, there is limited knowledge of the prevalence and incidence of COPD, patient characteristics and provider prescribing regimens in this region. Although the PRISA study is not representative of the total population in Latin American, it is, to our knowledge, the first population-based cohort study addressing knowledge gaps and informing public health policy-making on the impact of COPD in Argentina, Chile and Uruguay.

The close cooperation between experienced researchers in Argentina, Chile, Uruguay, and Tulane University in the USA is a study strength. Methodology to estimate prevalence is similar to that used in the PLATINO study, which will allow comparison of data between the Latin American countries studied in the PLATINO study and the baseline phase of the PRISA study. The PRISA study, however, has the advantage of being the first longitudinal study of pulmonary function in Latin America. In addition, questionnaires used in the HCHS/SOL study[19], a cohort study performed on Hispanic population in the USA will be also used in the PRISA study, allowing additional comparison between PRISA study subjects and the Latin American and Hispanic communities in the United States.

It is clear that chronic pulmonary diseases, including COPD, are leading causes of morbidity and mortality worldwide. Our study will provide important information about the epidemiology of COPD in select cities in Argentina, Chile and Uruguay. In the first phase of the study, it will describe COPD prevalence and associated risk factors, socioeconomic status, concurrent medical history, patient characteristics and physician prescribing trends. In the second phase, it will describe the decline in lung function over time, COPD incidence and any changes in patient characteristics and physician prescribing trends. This information is paramount to the development of appropriate public health legislation and preventive interventions.

## Competing interests

The authors declare that they have no competing interests.

## Authors' contributions

AR contributed to the conceptualization and design of the study and revised this manuscript critically. JH contributed to the conceptualization and design of the study. LB contributed to the conceptualization and design of the study and revised this manuscript critically. VI contributed to the conceptualization and design of the study and revised this manuscript critically. AL is participating in the analysis and interpretation of data and was involved in drafting and revising the manuscript. ES is participating in the acquisition, analysis and interpretation of data and was involved in the conception and design of the study and drafting the manuscript. FL contributed to the conceptualization and design of the study and is participating in the acquisition of data. JM contributed to the conceptualization and design of the study and is participating in the acquisition of data. MC contributed to the conceptualization and design of the study and is participating in the acquisition of data. HO contributed to the conceptualization and design of the study and is participating in the acquisition of data. PS contributed to the conceptualization and design of the study and is participating in the acquisition of data. JP contributed to the conceptualization and design of the study and is participating in the acquisition of data All authors gave final approval of the version to be published.

## Pre-publication history

The pre-publication history for this paper can be accessed here:

http://www.biomedcentral.com/1471-2466/11/34/prepub
